# Pyrazinamide-Induced Exfoliative Dermatitis in a Patient on Hemodialysis: A Rare Complication

**DOI:** 10.1155/2013/387293

**Published:** 2013-09-18

**Authors:** Krishnaswamy Jaisuresh

**Affiliations:** Department of Nephrology and Renal Transplantation, Assured Best Care Hospitals, No. 1 Malligai Salai, Annamalai Nagar, Trichy, Tamil Nadu 620 018, India

## Abstract

A 60-year-old male patient on maintenance hemodialysis was started on antituberculosis therapy with isoniazid, rifampin, ethambutol, and pyrazinamide for pulmonary tuberculosis. After 4 weeks of therapy, he developed pruritic lesions in the extremities followed by exfoliation. The lesions progressively spread over the entire body. Lesions resolved after withdrawal of antituberculosis medications and administration of oral corticosteroids and antihistamines. After 2 weeks antituberculosis drugs were rechallenged one at a time. Administration of oral pyrazinamide resulted in reappearance of symptoms (pruritis and erythema) within 48 hours. Pyrazinamide was substituted with ofloxacin while other three drugs were restarted without any side effects. The case illustrates a rare but potentially dangerous complication of pyrazinamide therapy.

## 1. Background 

Cutaneous adverse drug reactions (CADR) are often encountered with first-line antituberculosis therapy (ATT). Exfoliative dermatitis is a dangerous form of CADR which needs immediate withdrawl of all the four drugs. In a hemodialysis patient with active pulmonary tuberculosis, early withdrawl followed by prompt rechallenging to identify the causative agent and then to achieve cure of pulmonary tuberculosis is an interesting therapeutic challenge. 

## 2. Case History and Hospital Course

A 60-year male with three-week history of low-grade fever, weight loss, and sputum positive tuberculosis was started on thrice weekly antituberculosis therapy with isoniazid, rifampin, ethambutol, and pyrazinamide. He was on maintenance hemodialysis thrice a week (duration 5 hours) since 8 years and his routine medications included antihypertensives (amlodipine 10 mg per day, metoprolol 50 mg per day), erythropoietin, iron, folic acid, and phosphate-binding agents (calcium acetate). There was no change in prescription since last 6 months. 

ATT was administered in dosage of 150 mg of isoniazid, 450 mg of rifampicin, 400 mg of ethambutol, and 1000 mg of pyrazinamide. All drugs were given thrice a week, 24 hours prior to next dialysis. At the start of ATT his liver function tests (total bilirubin 0.6 mg/dL (0.1–1.3 mg/dL), direct bilirubin 0.4 mg/dL (0–0.5 mg/dL), total protein 5.9 g/dL (6–8.4 g/dL), albumin 3.8 g/dL (3.5–5.5 g/dL), SGOT 16 IU/l (5–40 IU/L), SGPT 18 IU/l (5–40 IU/L), alkaline phosphatase 24 U/L (35–150 U/L), LDH 120 IU/L (85–450 IU/L), uric acid 6 mg/dL (3.9–8.9 mg/dL), calcium 9.4 mg/dL (9–11 mg/dL), and inorganic phosphorus 4.3 mg/dL (2.5–4.5 mg/dL) were within normal limits. HBsAg, HIV, and anti-HCV antibody ELISA were negative. 

**Figure 1:**
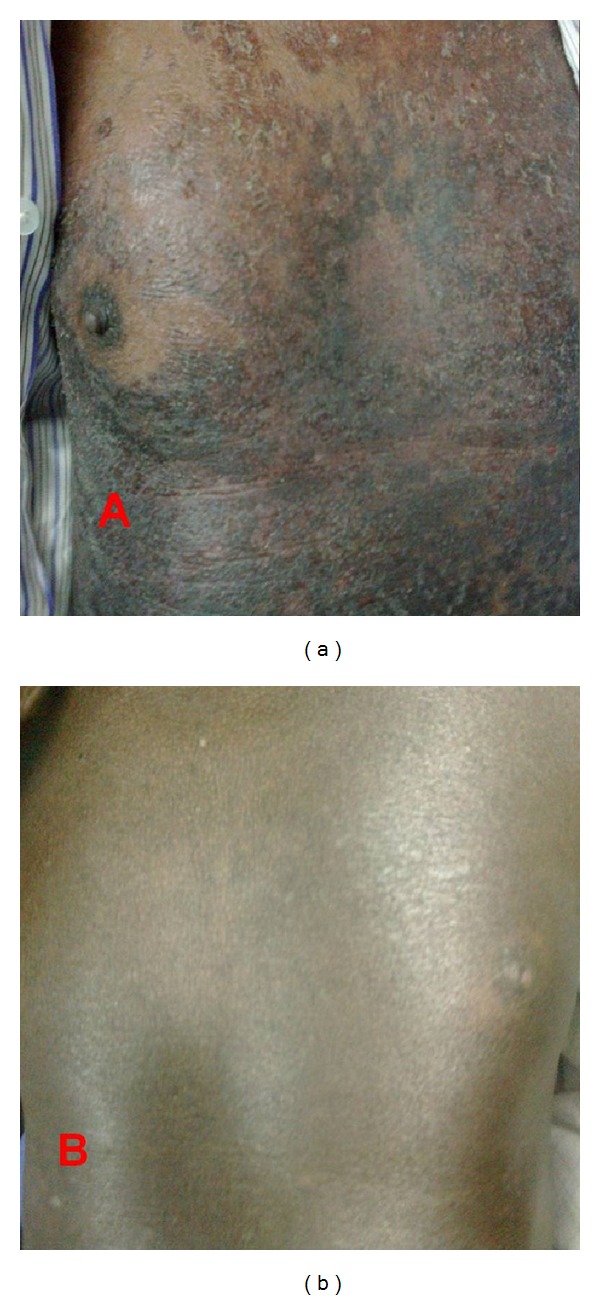
(a) Extensive exfoliation and hyperpigmentation on trunk after starting pyrazinamide. (b) Resolution of exfoliative lesions with residual hyperpigmentation 6 weeks after stopping PYZ.

After 4 weeks of ATT, he complained of pruritic erythematous lesions over both upperlimbs. The lesions gradually spread to involve the trunk and then the entire body. ATT was stopped immediately and antihistamines were administered. The lesions continued to spread followed by progressive exfoliation (Figure 1(a)). The patient developed alopecia and hypothermia. He was hospitalised and intravenous steroids were administered (IV methylprednisolone 40 mg per day for 3 days) along with antihistamines. The pruritis decreased initially followed by gradual decrease in erythema.

A complete reevaluation did not reveal any foci of viral infection. He denied using any prescriptions, over the counter drugs or herbal medications. Physical examination revealed pallor, pulse rate 92/min, blood pressure of 150/100 mmHg, temperature 36.9°C, and respiratory rate 15/min. Cardiac examination was unremarkable. The abdomen was soft and nontender and had no palpable hepatosplenomegaly. His investigations included hemoglobin 11.7 gm/dL, total leucocyte count 7.2 × 1000/microlitre (4–10 × 1000/microlitre) with eosinophils 12%, neutrophils 67%, lymphocytes 10% and monocytes 1%, platelet count of 262 cells/cmm (150–400 × 1000 cells/cmm), reticulocyte count 1.2%, serum creatinine 5.8 mg/dL (0.5–1.6 mg/dL), total bilirubin 1.2 mg/dL (0.1–1.3 mg/dL), direct bilirubin 0.7 mg/dL (0–0.5 mg/dL), total protein 5.7 g/dL (6–8.4 g/dL), albumin 3.5 g/dL (3.5–5.5 g/dL), SGOT 46 IU/L (5–40 IU/L), SGPT 48 IU/L (5–40 IU/L), and alkaline phosphatase 24 U/L (35–150 U/L). HBsAg, Anti-HCV, and HIV-ELISA were negative. The erythrocyte sedimentation rate 72 mm/hr was elevated. Ultrasound abdomen did not show any hepatosplenomegaly or lymphadenopathy. 

With a diagnosis of drug-induced exfoliative dermatitis, antihistamines and oral prednisolone (5 mg per day) were prescribed for next 5 days. 14 days after withdrawl of ATT he had significant resolution of constitutional symptoms with reduction in appearance of new lesions. ATT was restarted one at a time. Rifampicin (75 mg on day 1 followed by 300 mg on day 3) and then Isoniazid (50 mg on day 1 followed by 150 mg on day 3) were tolerated without any significant symptoms. Subsequently ethambutol (400 mg on day1and day 3) was administered without any side effects. When pyrazinamide 500 mg was readministered he developed pruritu and erythema associated with fever within 48 hours of administration. The symptoms resolved after antihistamine treatment for 3 days. The patient was continued on isoniazid, rifampicin, ethambutol, and ofloxacin thrice a week for next 8 weeks. Sputum reexamination for acid-fast bacilli was negative at the end of 8 weeks. Isoniazid and rifampicin were continued for next 4 months and stopped after sputum tested negative for acid fast bacilli. His liver function tests returned to normal limits after 4 weeks of pyrazinamide withdrawl and his skin lesions resolved completely during the next 6 weeks (Figure 1(b)).

## 3. Discussion 

Identification of causative agent is the most important challenge in patients with cutaneous adverse drug reactions (CADRs). Exfoliative dermatitis is a severe form of CADR which has been reported with all the four first-line antituberculosis drugs [1–3]. In patients with active pulmonary tuberculosis it is essential to use first-line agents to achieve cure of tuberculosis. Hence drug rechallenge is the only method to identify the causative agent. Though exfoliative dermatitis can be caused by various drugs and infections, temporal relationship between introduction of pyrazinamide and onset of CADR, resolution following withdrawl and the reappearance of symptoms when it was rechallenged, identified pyrazinamide as the causative agent. 

Pyrazinamide has been described to cause various skin reactions like maculopapular rash [4, 5], erythema multiforme [6], exfoliative dermatitis and DRESS syndrome [7]. Among the first line drugs pyrazinamide is the commonest cause of CADR (2.38%), followed by streptomycin (1.45%), ethambutol (1.44%), rifampicin (1.23%), and isoniazid (0.98%) [8]. It is not uncommon for exfoliative dermatitis to occur with more than one of the four drugs [9]. It is unclear whether renal failure predisposes to increased occurrence of CADR'S. So far no definite association exists between preexisting renal insufficiency and increased incidence of CADR'S8. The fact that symptoms appeared after 4 weeks of therapy is a learning point to be cautious about delayed side effects of these agents. 

From the limited experience of case reports, we conclude that exfoliative dermatitis is a uncommon potentially life-threatening form of CADR. Recovery and prognosis are favourable if ATT withdrawl and other supportive measures are initiated at the earliest.
